# MicroRNAs modulate neuroinflammation after intracerebral hemorrhage: Prospects for new therapy

**DOI:** 10.3389/fimmu.2022.945860

**Published:** 2022-11-01

**Authors:** Siqi Xia, Yonghe Zheng, Feng Yan, Gao Chen

**Affiliations:** ^1^ Department of Neurosurgery, Second Affiliated Hospital, School of Medicine, Zhejiang University, Hangzhou, Zhejiang, China; ^2^ Key Laboratory of Precise Treatment and Clinical Translational Research of Neurological Diseases, Zhejiang University, Hangzhou, Zhejiang, China

**Keywords:** microRNAs, intracerebral hemorrhage, neuroinflammation, mechanism, therapy

## Abstract

Intracerebral hemorrhage (ICH) is the most common subtype of hemorrhagic stroke. After ICH, blood components extravasate from vessels into the brain, activating immune cells and causing them to release a series of inflammatory mediators. Immune cells, together with inflammatory mediators, lead to neuroinflammation in the perihematomal region and the whole brain, and neuroinflammation is closely related to secondary brain injury as well as functional recovery of the brain. Despite recent progress in understanding the pathophysiology of ICH, there is still no effective treatment for this disease. MicroRNAs (miRNAs) are non-coding RNAs 17–25 nucleotides in length that are generated naturally in the human body. They bind complementarily to messenger RNAs and suppress translation, thus regulating gene expression at the post-transcriptional level. They have been found to regulate the pathophysiological process of ICH, particularly the neuroinflammatory cascade. Multiple preclinical studies have shown that manipulating the expression and activity of miRNAs can modulate immune cell activities, influence neuroinflammatory responses, and ultimately affect neurological functions after ICH. This implicates the potentially crucial roles of miRNAs in post-ICH neuroinflammation and indicates the possibility of applying miRNA-based therapeutics for this disease. Thus, this review aims to address the pathophysiological roles and molecular underpinnings of miRNAs in the regulation of neuroinflammation after ICH. With a more sophisticated understanding of ICH and miRNAs, it is possible to translate these findings into new pharmacological therapies for ICH.

## 1 Introduction

Intracerebral hemorrhage (ICH) is the most common subtype of hemorrhagic stroke. It is associated with high morbidity and mortality, accounting for 15% of all stroke cases and causing 50% of stroke-related mortality (1). Primary ICH accounts for 78–88% of all ICH cases. Its main causes include chronic hypertension and cerebral amyloid angiopathy (CAA). Secondary ICH results from diverse causes including aneurysm rupture, vascular malformation, and coagulopathy (2). Despite the various pathogeneses, the consequences of most ICH are devastating, and many patients suffer early death after occurrence or survive with disability (3). The incidence of ICH is increasing as a result of population aging and increased use of anticoagulants (4). However, effective therapeutics for ICH are still lacking, and the existing treatments are mainly supportive (5, 6).

Neuroinflammation is a major contributor to secondary brain injury and brain repair after ICH (7). Resident immune cells in the perihematomal regions are activated rapidly following blood extravasation from the vessels and release a series of toxic mediators into their environment, thus triggering an inflammatory cascade that evolves in a time-and space-dependent manner. Neuroinflammatory responses can cause not only instant local damage, but also long-term neurological dysfunction (8). Considering the important and double-edged effects of neuroinflammation, it would be beneficial to improve clinical outcomes by shifting the balance of its effects towards the beneficial side. Recently, numerous studies have aimed at alleviating inflammatory injury and promoting the reparative effects of neuroinflammation (9, 10). Several drugs have been tested in preclinical animal models or clinical trials, but their therapeutic effects are unsatisfactory (11–14). This is partly due to the lack of an explicit understanding of post-ICH neuroinflammation and technological limitations. Therefore, new perspectives to study neuroinflammation after ICH and new forms of drugs or interventional technologies may be required. MicroRNAs (miRNAs, miRs) are non-coding RNAs (ncRNAs) that bind to messenger RNA (mRNA) and silence target genes (15). Constituting a part of epitranscriptome, they have great significance in biological processes such as cell growth, differentiation, death, metabolism, and immune responses (16–19). Recently, many studies have found that miRNAs exert modulatory effects on central nervous system (CNS) inflammation. For example, miR-124 was discovered to induce microglia (MG) quiescence in the CNS as well as deactivate monocytes and macrophages. In addition, miRNAs have been found to regulate neuroinflammation after ICH by affecting multiple inflammatory components (20).

A better understanding of the influence of miRNAs on the brain and their interaction with the milieu is needed. This review summarizes miRNA modulations in the brain after ICH, divided by the important components of the brain immune system, including immune cells, inflammatory molecules, and pathways. It also describes the limitations and possible future directions for the study of miRNA functions and their therapeutic potential, which will pave a way for developing new therapeutics for this disease.

## 2 Pathophysiology of neuroinflammation after ICH

ICH refers to blood extravasation from vessels into the brain parenchyma, triggering a chain of damaging reactions. Hypertension-related ICH typically occurs in small thin-walled arteries from deep sites in the brain, such as the basal ganglia, thalamus, cerebellum, and brainstem (21), while CAA-related ICH is more often observed in lobal locations, especially at the junction of the cortical gray and white matter (11). Injuries after ICH can be divided into two types: primary and secondary (22). Primary brain injury after ICH is mainly due to stretching, compression, and deformation of the newly formed hematoma in the brain tissues. It can affect perihematomal blood flow and cause brain ischemia as well as elevate the overall intracerebral pressure and generate lethal brain herniation (23). Secondary brain injury develops soon after ICH and initially results from extravasated blood components. Its mechanisms involve, but are not limited to, the hemostasis response, neuroendocrine axis activation, neuroinflammation, cell death, thrombin toxicity, oxidative stress, erythrocyte lysis, excitotoxicity, and brain edema (24). Among these, neuroinflammation is recognized as the most prominent contributor to secondary brain injury and is often associated with poor clinical prognosis.

The post-ICH brain environment is a dynamically changing milieu with many inflammatory components, including immune cells, inflammatory mediators, and intricate signaling pathways. They interact with each other and contribute to the course of inflammation in a combined manner (25). The main immune cells are microglia, astrocytes, neutrophils, T lymphocytes, and mast cells, which reside in the brain or infiltrate from the peripheral blood (9). Inflammatory mediators include cytokines, chemokines, complements, proteases such as matrix metalloproteinases (MMP), prostanoids, reactive oxygen species (ROS), inducible nitric oxide synthase (iNOS), blood-derived thrombin and plasmin, and other toxic substances (26, 27). Early after blood components enter the brain parenchyma, immune cells bearing pattern recognition receptors (PRRs) such as Toll-like receptors (TLRs) can detect injurious substances and trigger a cascade of inflammatory reactions in the perihematomal regions (28). These inflammatory responses include, but are not limited to, immune cell activation, peripheral immune cell infiltration such as neutrophils and macrophages, release of diverse inflammatory mediators, and activation of diverse molecular pathways. They aggravate inflammation and damage cells in the brain microenvironment. In turn, damage-associated molecular patterns (DAMPs) from injured cells can aggravate neuroinflammation (29). The DAMPs include adenosine, heat shock proteins (HSPs), high-mobility group box 1, interleukin-33 (IL-33), hyaluronan, heparin sulfate, amyloid-beta, oxidized low-density lipoprotein, peroxiredoxins, and mitochondrial-derived N-formyl peptides (23, 24, 30). Thus, early activation of microglia and other immune components converts the homeostatic non-inflammatory CNS parenchyma into an abundant pro-inflammatory milieu. After some time, with reparative microglia and astrocytes taking dominance, the pro-inflammatory milieu gradually transforms to a reparative milieu that promotes neurogenesis and anti-inflammatory reactions (31, 32). Throughout the process, some important signaling pathways exist, such as the nuclear factor-κB (NF-κB) pathway that promotes the transcription of diverse pro-inflammatory genes (33). They have some similar and unique characteristics in different cells or different phases, and can work at the cellular, intercellular, or even whole brain levels.

Generally, immune cells in the post-ICH milieu can be divided into pro-inflammatory and anti-inflammatory types. The former contains microglia, astrocytes, neutrophils, and Th1 cells. They can adapt to the immune response in the brain and release an array of pro-inflammatory mediators that damage cellular integrity, extracellular matrix stability, and blood-brain barrier (BBB) permeability (34). The latter includes microglia, astrocytes, CD4+ T cells, activated Treg cells, Th2 cells, Breg cells, and regulatory dendritic cells (35). They contribute to hematoma removal, brain debris clearance, inflammation resolution, and tissue regeneration (36). It is noteworthy that many components of the brain immune system have been found to have various subtypes according to different determinants and contexts, such as microglia and astrocytes (37–39). Microglia and astrocytes can have opposite functions in different states. This implies the intricacy and flexibility of brain inflammatory components, as well as the complexity of the overall neuroinflammation course which has both detrimental and beneficial effects (12, 40).

Apart from local brain inflammation, imaging evidence has shown that there is global brain inflammation in regions remote from the primary injury sites after ICH. It shows a unique temporal evolution and may be relevant to clinical outcomes, such as dementia and cognitive decline (41). Mechanistically, it occurs likely by inflammatory components spreading through the extracellular space or cerebrospinal fluid, cytokines transmitting signals, chronic systemic mediators such as C-reactive protein increasing, endothelial cells interacting with activated platelets or leukocytes that cause vascular inflammation, and white matter mechanical propagation, diaschisis, or other forms of damage (such as DAMPs and released mediators) (42).

In summary, post-ICH neuroinflammation is an evolving and overlapping process, initiated rapidly after ICH occurrence, progressing over days and even weeks, and providing different molecular targets for treatment in different time windows.

## 3 microRNA biogenesis and functions

### 3.1 microRNA biogenesis

miRNAs are highly conserved, 17-25 nucleotides long, single-stranded ncRNAs transcribed from the genome (43). They play an important role in post-transcriptional gene regulation by binding to mRNA sequences (44, 45). miRNAs are encoded as either intragenic or intergenic in nature. Intragenic miRNAs can be transcribed from individual monocistronic miRNA genes, polycistronic miRNA clusters, or introns of protein-coding genes. To date, more than 1000 miRNAs are known to be expressed in the human body (46). The canonical generation of miRNAs starts with the formation of pri-miRNA, a double-stranded RNA containing approximately 300–1000 nucleotides with a loop structure (47). It is catalyzed by RNA polymerase II or III in the nucleus. It is then processed by the microprocessor complex containing the enzymes Drosha and DGCR8 to produce a pre-miRNA, a stem-loop structure approximately 70–90 nucleotides long (48). Subsequently, the pre-miRNA is exported from the nucleus to the cytoplasm by exportin-5 and Ran-GTPase on the nuclear membrane (49). The cytoplasmic RNase III-like endonuclease Dicer enzyme and TRBP cleave the pre-miRNA into a miRNA duplex without a loop; later, it is unwound by helicase to form single-stranded miRNAs that are approximately 22 nucleotides long (47, 50). While the passenger strand of the miRNA duplex undergoes degradation and forms processing bodies (P-bodies), the other strand becomes mature miRNA (47, 51). It incorporates argonaute (Ago) protein 2 to form an RNA-induced silencing complex (RISC). In addition to the canonical pathway, several noncanonical pathways of miRNA biogenesis have been identified, such as microprocessor-independent and Dicer-independent methods (47).

### 3.2 microRNA functions

miRNAs exert their functions mainly by interacting with mRNA. First, the 2–7 nucleotide seed sequence in miRNA binds to mRNA with perfect or imperfect complementarity, usually in the 3’ untranslated region (UTR) of mRNA (52). Then, miRNAs suppress protein levels with the aid of the TNRC6 (GW182) protein *via* either translational repression, mRNA destabilization, or sequestration in P-bodies (53). Destabilization is the most common type of steady-state cell. The degradation of mRNA or endonucleolytic cleavage can occur when the complementarity between miRNA and mRNA is high (15). The mechanism of mRNA destabilization includes: i) Deadenylation: shortening of the poly(A) tail in miRNAs by deadenylase complexes CCR4–NOT and PAN2–PAN3, and subsequent mRNA decay resulting from oligouridylation of deadenylated mRNAs by TUT4/7. The TNRC6 protein also leads to the dissociation of poly(A)-binding protein, thus promoting the deadenylation process (54). ii) Decapping: The CCR4–NOT complex removes the 5’ m7G cap from the mRNA *via* the decapping enzyme DCP2 upon activation by the decapping activator DDX6. iii) 5ˊ to 3ˊ exonucleolytic decay: XRN1 leads to 5’-3’ endonuclease degradation (55).

**Figure 1:**
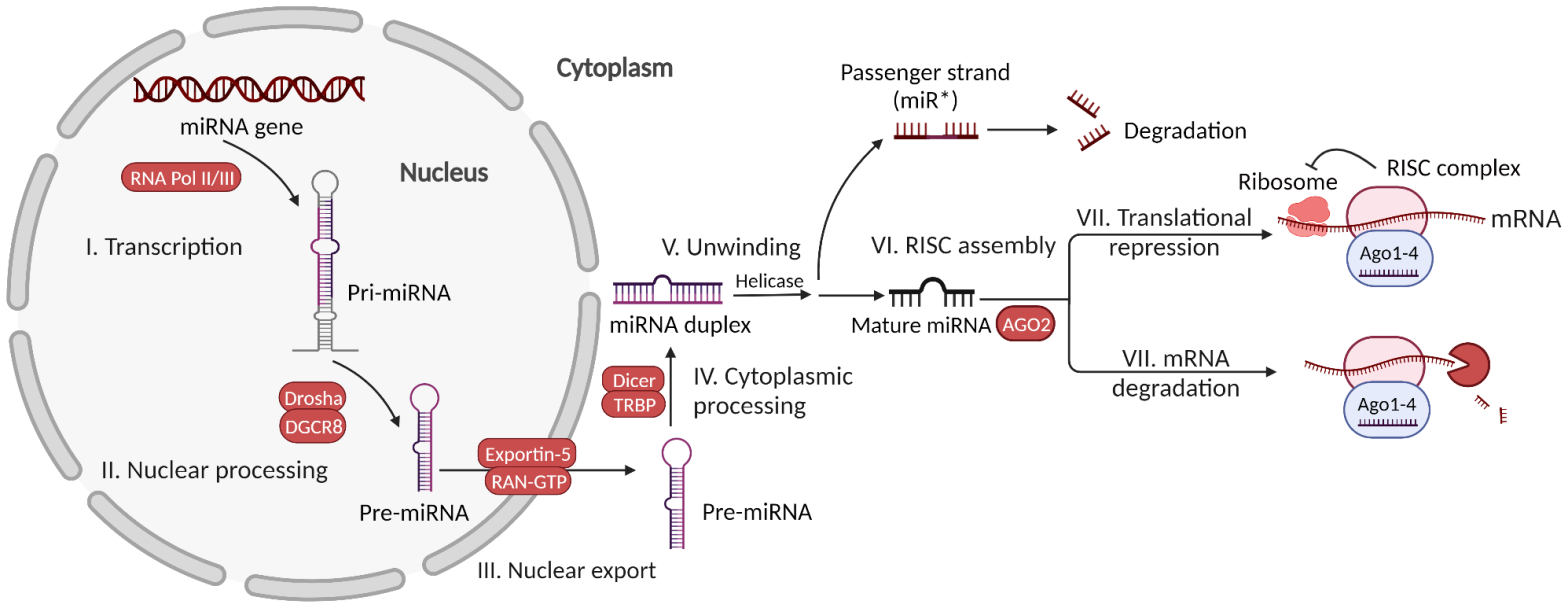
MicroRNA biogenesis and functions. Canonically generated miRNAs go through transcription of the miRNA gene, nuclear processing of pri-miRNA, nuclear export of pre-miRNA, cytoplasmic processing of pre-miRNA, miRNA duplex unwinding, and assembly with AGO2 to form the RISC complex. Then, the mature miRNA in the RISC complex can exert functions *via* either mRNA translational repression or mRNA degradation.

Translational repression usually occurs when miRNAs and mRNA have imperfect complementarity and can function at any stage of translation including initiation, elongation and termination, as well as co-translational degradation (56). It has relatively weaker effects than destabilization, is always accompanied by destabilization, and occurs much faster (within 30 min) than mRNA decay (60 min) after binding to mRNAs (57). Global analysis of miRNA function shows that only 6–26% repression of each target in human cells results in pure translational repression without mRNA decay. Using the ribosome profiling method, it was found that miRNAs inhibit cap−dependent translation at translation initiation; however, the precise mechanism remains elusive (58). In particular, in cellular conditions such as serum starvation, miRNAs have been found to promote the translation of certain mRNAs (47). Recently, *in situ* single-molecule imaging has revealed that miRNAs are more likely to bind to translated mRNAs than to untranslated mRNAs, and this binding occurs immediately after nuclear export (59). One individual miRNA can target tens or even hundreds of mRNAs and thus can regulate an entire pathway, whether overexpressed or underexpressed. mRNAs can be targeted by specific abundant miRNAs or multiple low-abundance miRNAs, depending on their characteristics (60). Genes that are regulated by multiple miRNAs show a distinct reduction in protein expression noise (61). The specific mechanism of miRNA function is complicated, and many debates still exist regarding some facets, such as subcellular compartmentalization (62, 63). Here is the summary of miRNA biogenesis and functions (**Figure 1**).

### 3.3 microRNAs in CNS

Studies have shown that miRNAs can shape the cellular phenotypes in the human body. A small number of highly abundant miRNAs constitute up to half of the miRNAs in the RISC in cells (64). After ICH, the temporospatial expression profile of miRNAs in neural cells and tissues is altered in a certain way in response to the damage such as inflammation. To some extent, this unique change can be reflected by peripheral miRNA expression since miRNA can be released into the bloodstream (65, 66). Some studies have focused on the potential of using miRNAs as biomarkers in diseases affecting the CNS, such as ICH, Alzheimer’s disease, ischemic stroke, amyotrophic lateral sclerosis, and epilepsy (67, 68). However, since there are multiple sources of circulating miRNAs, it can be difficult to determine whether the miRNAs are from the injured brain, peripheral sites, or both.

In addition, many studies have shown that miRNAs function in both homeostatic and pathological states of the CNS (69–71). For example, miR-124, a neuron-specific miRNA, was found to play a significant role in neuronal maturation, differentiation, and survival during normal CNS development (72). In the diseased state, abnormal miRNA expression can modulate the inflammatory reactions within the brain. After ICH, large-scale gene expressions are altered instantly due to the local brain damage, mechanistically by the genetic modulation of transcriptional factors, epigenetic DNA methylation and histone modification, and post-transcriptional miRNA regulation (9). Although some alterations might merely be the result of the injury with no practical boost in worsening the outcome, others result in disturbances of the molecular composition and cellular features of brain cells and consequently affect neurological functions (73, 74). miRNAs, with much evidence, fall into the latter type, being expressed aberrantly and having effects on brain functions after ICH. It can modulate neuroinflammation by affecting prominent inflammatory components, including immune cells, molecules, and pathways, indicating its potential as a therapeutic target in ICH (48, 75,). This review focuses on the typical inflammatory responses that have been mainly studied in every previous report. To avoid overlapping, most studies were categorized into only one subtype.

## 4 Mechanism of microRNA in modulating neuroinflammation after ICH

### 4.1 microRNAs and immune cells

#### 4.1.1 Microglia

Microglia fulfil important homeostatic functions but can also promote or participate in neuroinflammation following brain injury. Early after ICH, brain-resident microglia and macrophages that flood in from the blood are activated by the leaked blood components, and adopt different states from the original, in which they perform specialized functions (9, 76, 77). Regarding microglial states, the once widely used “M1/M2” dichotomy has been discarded by the field, because dividing activated microglia into two distinct phenotypes is an oversimplification and is not consistent with the heterogeneity of microglia in health or disease context (38). The precise definitions of microglial states have been elusive (78–80). Researchers think that it should take account microglial intrinsic and extrinsic elements (i.e., species, sex, genes, pathogens), spatiotemporal context in the brain, and layers of complexity (i.e., epigenomic, transcriptomic, proteomic) when defining microglial states (38).

Studies have shown that some miRNAs regulate microglial activities by modulating signaling pathways and exerting positive functions after ICH. A study has shown that miR-21/miR-146a alleviates microglia-mediated inflammatory responses in post-ICH rats (81). Likewise, miR-181b mimics significantly decreased microglial infiltration and HSP family A (Hsp70) member 5 (HSPA5), NF-κB p65, IL-6, IL-1β, and tumor necrosis factor (TNF)-α levels in perihematomal tissue of ICH mice (82). Mechanistically, the TLR4/NF-κB pathway is the major signaling pathway that mediates pro-inflammatory cytokine production (28). There is evidence that NF-κB translocates into the nucleus and promotes the transcription of inflammatory mediators including cytokines following brain injury. miR-7 was found to attenuate inflammation in both *in vivo* and *in vitro* models by inhibiting TLR4 (83). miR-367 attenuates NF-κB activation and pro-inflammatory mediator production after ICH in cultured microglia and in a mouse model by suppressing IL-1 receptor-associated kinase (IRAK) 4 (84). In addition, an *in vitro* study demonstrated that tumor necrosis factor receptors (TNFR)-dependent aggravation of neuroinflammation in a thrombin-induced human microglia model is partly attributable to the decrease in miRNA-181c and its downstream regulation of MLL1 and NF-κB, implicating its role in post-ICH neuroinflammation (85). Apart from that, miR-26a overexpression significantly reduced microglia-induced pro-inflammatory cytokine production in an ICH mouse model. *In vitro* experiments revealed that the overexpression of high mobility group A (HMGA) 2, a verified target of miR-26a, reverses the miR-26a-mediated inflammatory response, suggesting its important role in this process (86). HMGA2 is a member of the HMGA family and is a chromatin-associated protein that participates in cell proliferation, differentiation, tumorigenesis, and inflammation (87). Besides, miR-183-5p decreases microglial survival and activation following inflammation and oxidative damage after ICH in mice, likely by inhibiting heme oxygenase (HO)-1 independently of nuclear factor erythroid 2-related factor 2 (Nrf2), a well-known mediator of oxidative stress responses (88). Administration of exosomes derived from bone mesenchymal stem cells (BMSCs-Exos) enriched with miR-146a-5p through the tail vein reduces neuroinflammation and exert positive effects after ICH by inhibiting IRAK1 and nuclear factor of activated T cells 5 (NFAT5) (89), which have been verified to play roles in inflammatory responses. miR-124 alleviated inflammatory damage both *in vivo* and *in vitro* by inhibiting CEBP-α, a member of the CEBP transcription factor family, which promotes the differentiation of myeloid progenitor cells into granulocytes, including monocytes and macrophages (90). Similarly, an *in vitro* study verified that miR-367 reduced apoptosis after erythrocyte lysate-induced damage by downregulating CEBP-α (91). Apart from these, Let-7a alleviated the inflammatory response in an ICH mouse model by downregulating casein kinase 2 interacting protein-1 (CKIP-1), a newly discovered regulator of macrophage proliferation (92). miRNA-575 inhibits phosphatase and tensin homolog (PTEN), thus playing a significant role in l-lysine-mediated alleviation of neuronal injury after ICH (93).

Other miRNAs play detrimental roles in neuroinflammation after ICH by regulating microglia. miR-222 increases microglia-mediated pro-inflammatory cytokine production and brain water content in an ICH mouse model by downregulating integrin subunit beta 8 (ITGB8), an integrin previously verified to play a vital role in brain vessel homeostasis (94). The autophagy process after ICH can be modulated by miRNAs, which affect neuroinflammation. miR-144 targets mammalian target of rapamycin (mTOR) by directly interacting with its 3’-UTRs, thus inhibiting autophagy in hemoglobin-treated microglia, consequently promoting an inflammatory response in microglia (95). In contrast, another study reported that miR-144 promotes IL-6, IL-1β, and TNF-α levels and microglial autophagy *via* mTOR, thus aggravating brain edema and neurological deficits in mice after ICH (96). Overexpression of miR-23b suppresses MG activation, neuronal apoptosis, and neurological deficits in ICH rat models. It also alleviates inflammation of BV2 cells and apoptosis of co-cultured HT22 cells under hemin stimulation *in vitro*, at least partly by targeting inositol polyphosphate multikinase (IPMK), thus inhibiting the AKT (protein kinase B, or PKB)/mTOR autophagy pathway (97). The different results of these studies reflect the duality or controversy regarding the role of the autophagic pathway in microglia after ICH: whether it is beneficial, detrimental, or both (40). Besides, miR-34a-5p overexpression by agomiR-34a-5p injected into the lateral ventricles accelerates neuronal apoptosis in ICH rats by negatively targeting Krüppel-like factor 4 (Klf4), a zinc-finger-containing protein that plays an important role in many physiological processes (98). Another study found that miR-494 promoted inflammatory injury *in vitro* and *in vivo* after ICH by inhibiting Nrdp1 and its downstream transcription factor CEBP-β (99). Through miRNA-seq and mRNA-seq, miR-181a was found to be the most highly connected node in the miRNA-mRNA interconnection network that regulates differentially expressed genes after ICH. miR-181a, monocytes, and IL-8 form an interconnected network that causes neuroinflammation in the post-ICH milieu (100). This indicates the importance of further studies on the interconnectome associated with miRNAs. Since the effects of microglia on the course of inflammation are correlated with time, modulation of microglial activation should be performed more precisely at the most appropriate time in the disease course.

#### 4.1.2 Astrocytes

Astrocytes are the most abundant, complex, and largest glial cells in the brain. They participate in various physiological processes, including neurotrophy, synaptogenesis, BBB maintenance, and metabolism. After ICH, they are involved in peripheral immune cell recruitment, secretion of toxic mediators, interaction with microglia, and later-stage brain tissue repair (39, 101, 102). miRNAs have been found to regulate their functions. In a hemin-induced hemorrhagic astrocyte model, phosphatidylinositol-3-kinase (PI3K)/Akt pathway inhibitors suppressed miRNA-146a-3p, thus elevating aquaporin 4 (AQP4) expression and alleviating inflammatory injury (103). Dexamethasone-mediated alleviation of neuroinflammation in post-ICH mice is associated with a reduction in miR-155 and an increase in suppressor of cytokine signaling (SOCS-1) expression. Additional experiments have demonstrated that miR−155 inhibits SOCS−1 expression in astrocytes, suggesting that miR-155 might regulate astrocyte inflammatory functions by targeting astrocytic SOCS-1 (104). However, studies on miRNAs and astrocytes after ICH are scarce, and further exploration is required in this field.

#### 4.1.3 Leukocytes

After ICH, peripheral leukocytes infiltrate the damaged BBB into the CNS, a process that is promoted by chemokines secreted mainly from microglia and astrocytes. The major leukocytes contributing to neuroinflammation include neutrophils, lymphocytes, mast cells, monocytes, and dendritic cells. Among these, neutrophils were the most prominent type. Neutrophils recruited from the peripheral blood are located in the brain parenchyma and secrete many toxic mediators, such as pro-inflammatory cytokines (e.g., TNF-α), MMP-9, and ROS, which activate other cells and exacerbate secondary brain injury. Moreover, neutrophils can recruit macrophages to phagocytose hematomas and brain debris (105). Growing evidence has shown that neutrophils can be regulated by miRNAs after ICH. Inhibition of let7c by antagomir decreases perihematomal inflammation including neutrophil infiltration and microglial activation 3 days after ICH in rats, at least partly by activating the insulin-like growth factor 1 receptor (IGF1R) pathway (106). miR-183-5p alleviates early inflammation including neutrophil infiltration in the hemorrhagic striatum of mice after ICH by downregulating HO-1 (88). Administration of miR-146a-5p-riched BMSCs-Exos *via* the tail vein 24 h after ICH noticeably reduced neutrophil infiltration and alleviated the inflammatory response in injured brain tissues of rats (89).

T lymphocytes are the main cellular components of the adaptive immune system within the brain after ICH (24). Studies have found that T cells can patrol the cerebrospinal fluid in the healthy CNS to conduct immune surveillance (107). And after ICH, activated T cells can cross the BBB and contribute to brain injury through a number of mechanisms such as microvascular dysfunction, neuronal apoptosis, inflammatory molecules production, neutrophil recruitment, and interaction with microglia (108). The major subtypes of T cells include CD4+ Th cells (including Th1, Th2, and Th17 cells), CD8+ cytotoxic T cells, memory T cells, and T-regulatory (Treg) cells. They have different molecular expression profiles and cellular functions, with some mainly exerting injurious effects and others being reparative after ICH. Among them, CD4+ T cells are the predominant populations in the post-ICH brain milieu (108). Studies suggest that many miRNAs can serve as positive or negative regulators of T cell development and function, exemplified by the fact that models with certain miRNA deficiency can have CD4+ and CD8+ T cell dysfunction (109). Mechanistically, miRNAs can regulate T cell activation by modulating its key molecules and pathways, such as cell survival and signaling molecules (e.g., BIM, BCL-2, cell cycle regulators, mTOR), membrane receptors (e.g., ICOS, CD28, CTLA-4, PD-1), cytokines (e.g., IL-2, IFN-γ, IL-10, TGF-β), kinases (e.g., PI3KR1), and phosphatases (e.g., TCR Inhibitory phosphatases, PTEN) (110). However, there are few studies regarding miRNA regulating T cell functions in the post-ICH context by far. Therefore, the effect of miRNA on different T cell subtypes after ICH needs further exploration.

Mast cells are granulocytes that reside in the normal brain and release toxic mediators, recruit macrophages, and interact with microglia and astrocytes upon activation (111). Their degranulation or activation exacerbates brain damage, while their stabilization or deficiency reduces damage after ICH. Notably, miRNAs can modulate their functions. Inositol-requiring enzyme 1 (IRE1) α, a sensor signaling protein related to endoplasmic reticulum stress, promotes mast cell degranulation and brain damage after ICH in mice, likely by downregulating Lyn kinase. miR-125b-2-3p is involved in this process, suggesting its role in the regulation of mast cell function (112). However, further studies are required to elucidate these detailed mechanisms.

### 4.2 microRNAs and molecular pathways

#### 4.2.1 NF-κB pathway

NF-κB is the most prominent transcription factor that aggravates the inflammatory response following ICH. Following ICH, it translocates into the nucleus and promotes transcription of an array of inflammatory mediators, including cytokines, chemokines, enzymes such as iNOS and cyclooxygenase-2 (COX-2), cell receptors, and adhesion molecules (113). An *in vivo* experiment showed that miR-195 inhibits NF-κB signaling, including IκKa and phosphorylated inhibitors of κB p-IκBa/b, reduces ubiquitin-dependent IκB degradation, and leads to suppression of p65 (RelA)/p50 and RelB/p52 nuclear translocation. Its anti-inflammatory effect was confirmed by observing decreased TNF-α, IL-1b, IL-6, and monocyte chemoattractant protein-1 (MCP-1) in rat brains (114). Likewise, miR-146a plays a role in long non-coding RNA (lncRNA) MALAT1 knockdown conferred neurological protection against ICH in rats, including the alleviation of neuroinflammation, oxidative stress, and brain cell apoptosis, probably by regulating NF-κB (115). TLR4-mediated NF-κB signaling has long been found to participate in the pathogenesis of secondary brain injury after ICH. Inhibiting miR-93 by injecting miR-93 antagomir through the lateral ventricle attenuates neuroinflammation, cerebral edema, neuronal apoptosis, and improves neurological function in ICH rats by suppressing the TLR4/NF-κB pathway (116). Furthermore, the myeloid differentiation factor 88 (MyD88)/TRIF (TIR domain-containing adaptor inducing interferon-β) signaling pathway has been implicated in the activation of NF-κB after ICH (117). For example, a study found that miR-140-5p overexpression attenuates pro-inflammatory cytokine production and apoptosis in the perihematomal striatum following ICH in rats, at least partly by suppressing TLR4 and the downstream MyD88/TRIF/NF-κB inflammatory pathway (118). Together, these studies indicate the important role of miRNAs in NF-κB and associated signals. However, further studies are required to consider the multiplicity of NF-κB-related pathways.

#### 4.2.2 NLRP inflammasomes

Inflammasomes are a kind of PRR composed of multiprotein complexes that serve as a platform for caspase-1 activation, IL-1β, IL-18 release, and pyroptosis. NLRP3 (Nucleotide-binding oligomerization domain-like receptor [NLR] family pyrin domain-containing 3) is a member of the inflammasome family and a sensor of innate immune cells. It can be activated by DAMPs or pathogen-associated molecular pattern (PAMPs) and promotes the production of pro-inflammatory cytokines IL-1β and IL-18 (119). There have been reports that NLRP3 plays a vital role in post-ICH inflammation, and it can be regulated by miRNAs in central nervous diseases such as prosopalgia. In ICH, miR-223 mimics have been found to alleviate inflammation and neuronal injury in erythrocyte lysis-induced microglia by targeting NLRP3. *In vivo* experiments also demonstrated that miR-223 reduces inflammation in ICH mice (120). miR-152 suppresses post-ICH neuroinflammation and neuronal death both *in vivo* and *in vitro* by inhibiting thioredoxin interacting protein (TXNIP)-mediated NLRP3 activation (121). Tumor necrosis factor receptor-associated factor 6 (TRAF6), an intracellular scaffold protein, has been shown to participate in inflammatory signal transduction pathways of the TNF, IL-1R, and TLR receptor families. It was also found to be involved in the transcription-independent activation of NLRP3, but whether it regulates NLRP3 in the post-ICH milieu was not mentioned (122). A previous study reported that overexpression of miR-194-5p inhibits the activation of NLRP3 by inhibiting TRAF6 binding to it, thus alleviating ICH-induced neuroinflammation in rats (123). Correspondingly, an *in vitro* study found that miR-124-3p overexpression using its mimics distinctly inhibited neuroinflammation and apoptosis in lipopolysaccharide (LPS)-induced human microglial HMC3 cells by inhibiting TRAF6 and NLRP3 (124). Moreover, the TRAF6/NF-κB pathway is implicated in ICH damage. A study discovered that miR-146a mimics administered by intracerebroventricular injection reduced pro-inflammatory cytokine expression, brain edema, neuronal apoptosis and oxidative stress, at least partly by suppressing the TRAF6/NF−κB pathway in ICH rats (125). In addition, miR−183−5p enriched BMSC-extracellular vesicle (EVs) delivered *via* the tail vein alleviated neuroinflammation after ICH in diabetic rats, as well as reduced inflammatory factor production in hemoglobin-hemin BV2 cells; notably, these effects could be reversed by inhibiting miR-183-5p in EVs. Mechanistically, these effects are likely due to miR-183-5p downregulating programmed cell death 4 (PDCD4) and subsequently suppressing NLRP3 (126). Concordantly, a previous study demonstrated that miR-340-5p alleviates inflammatory responses, brain water content, and neurological deficits in ICH rat models. It also repressed LPS-induced inflammation in cultured microglial cells, likely by targeting PDCD4. However, the concrete role and mechanism of action of PDCD4 in neuroinflammation remains to be verified (127).

Apart from NLRP3, NLRP6 and NLRP1 also have regulatory effects on post-ICH injury. There are contradictory views regarding the function of NLRP6 after ICH. One study found that upregulation of NLRP6 in perihematomal brain tissues alleviates ICH-induced brain injury, including inflammation (128), whereas another study showed that NLRP6 promotes neuroinflammation after ICH by activating autophagy in an inflammasome-dependent manner (129). Moreover, miR-331-3p overexpression by injected mimics aggravated ICH-induced neuroinflammation in both a mouse model and hemin-treated BV2 cells by suppressing NLRP6 (130). Despite these findings, additional studies are warranted to elucidate the specific mechanisms and to consider NLRP inflammasomes, especially NLRP3, as therapeutic targets for miRNAs after ICH.

#### 4.2.3 PI3K/AKT pathway

The PI3K/PKB pathway plays a vital role in various physiological processes, including cell growth, proliferation, survival, metabolism, neurite extension, and synaptic plasticity. It is also found to regulate inflammation after the development of brain diseases, including Parkinson’s disease and pneumococcal meningitis, as well as after physiological aging (131). Recently, a series of studies have verified its role in ICH pathogenesis and its interactions with downstream molecules, such as NF-κB, Nrf2, and glycogen synthase kinase 3β (GSK3β) (132–134). Research has shown that miR-181b inhibitors promote neuroinflammation and oxidative stress and aggravate neuronal apoptosis and caspase-3 activity, thus exacerbating brain edema and neurological damage in ICH rats, probably by regulating the PI3K/AKT pathway. Inhibition of miR-181b obviously reduced p-AKT protein levels and p-AKT/AKT ratios compared to sham groups, suggesting that regulation of this pathway occurred (135). However, since the PI3K/AKT pathway’s main function is to regulate cell survival and death, it remains to be elucidated as to whether it affects the course of neuroinflammation directly or indirectly by manipulating immune cell survival.

#### 4.2.4 Nrf2 pathways

NRF2 is a transcription factor that regulates cellular detoxification and antioxidant responses by regulating gene expression. After ICH, it promotes antioxidative and anti-inflammatory responses, microglial phagocytosis, and hematoma dissolution (136). miRNAs have also been found to influence Nrf2. The miRNA-155 inhibitor antagomir-155 increases the expression of brain and muscle Arnt-like protein 1 (BMAL1) and activates the Nrf2 signaling pathway to attenuate neuroinflammation, oxidative stress, and neuronal death after ICH in rats (137). In addition, the miRNA-139/Nrf2/NF-κB axis may play a critical role in monomethyl fumarate’s (MF) role in conferring alleviation of neuroinflammation and oxidative stress, particularly considering that miR-139 overexpression reduces nuclear NF-κB levels and elevates nuclear Nrf2 levels in SH-SY5Y cells compared to controls and that levels of all three molecules are altered by MF treatment (138). However, further exhaustive studies are required to confirm this adjustment.

#### 4.2.5 Other molecular pathways

In addition to the molecules mentioned above, miRNAs have pivotal roles in the pathogenesis of post-ICH inflammation, along with some other targets. For example, miR-132 overexpression in ICH mice decreased the number of microglia and inflammatory cytokines in the perihematomal region, reduced BBB permeability, ameliorated brain edema, alleviated neuronal apoptosis, and attenuated neurological deficits, likely by inhibiting acetylcholinesterase (AChE) expression, thus promoting the anti-inflammatory effect of acetylcholine (ACh) (139). Overexpression of miR-590-5p noticeably reduces inflammatory cytokine production and improves brain edema and neurological functions in ICH mice, at least partially by inhibiting the expression of Pellino-1 (Peli1), a gene that has been reported to be abundantly expressed in microglia and promotes microglia-mediated inflammation (140). And inhibition of miR-21-5p alleviates inflammatory injury, including microglial activation, pro-inflammatory factor production, brain edema, neuronal apoptosis, and hematoma formation after ICH, thus attenuating neurological deficits by regulating the phosphorylated extracellular signal-related kinase (p-ERK)/HO-1 pathway, which is involved in the oxidative stress responses (141). Genetic knockout of miRNA-144/451 promotes inflammatory responses and oxidative stress, thus aggravating hemin-induced brain injuries in ICH mouse models by regulating the transcriptional factor FoxO3/14-3-3 axis (142). AntagomiR-23a-3p reduces ICH-induced pro-inflammatory cytokines such as IL-6, IL-1β, and TNF-α, inhibits neuronal ferroptosis, and facilitates nuclear factor E2-like 2 (NFE2L2) nuclear translocation in rat ICH models (143). In addition, miRNAs have been found to interact with lncRNAs in some brain diseases, such as ischemia, glioma, depression, and Parkinson’s disease. lncRNA Mtss1-promoted inflammatory injury in ICH mouse models was associated with a reduction in miR-155, the direct target of Mtss1 in cerebral tissues, implicating the positive role of miR-155 in neuroinflammation by interacting with lncRNA Mtss1 (144). However, another study has the opposite view of miR-155, showing that intracerebroventricular infusion of miR-155 inhibitor, but not its scramble, attenuated pro-inflammatory cytokines and oxidative stress biomarkers and elevated vascular endothelial growth factor (VEGF) expression in the parietal cortex and hippocampus of ICH rats (145). Therefore, due to the scarcity of associated literature, more studies are required to further verify the explicit and complete role of miRNAs in these molecules.

## 5 Prospects for new therapies

### 5.1 Feasibility of using microRNAs as therapeutic targets for treating neuroinflammation after ICH

**Figure 2:**
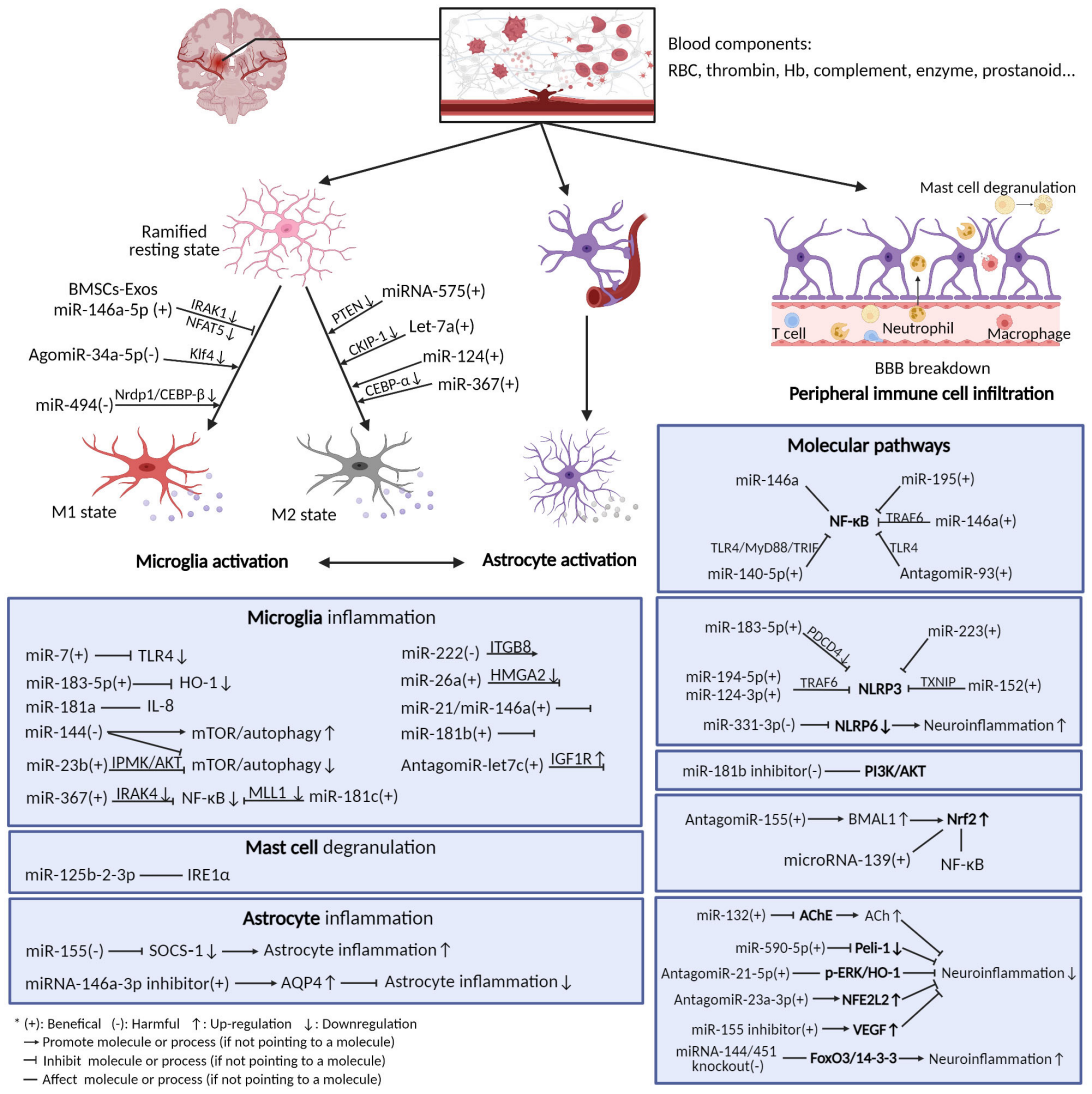
Neuroinflammation after ICH and microRNAs’ modulation of it. After ICH, blood components extravasated from ruptured vessels activate resident immune cells in the brain (mainly microglia and astrocytes), thus triggering neuroinflammation. The immune cells release a variety of inflammatory mediators and activate diverse molecular pathways. Many miRNAs can exert functions on these cells and pathways.

Here is the summary of the literature pertaining to the effect of miRNAs on post-ICH neuroinflammation, categorized according to the different immune cells, molecules, and pathways involved (**Figure 2**, **Table 1**). Most miRNAs have either positive or negative effects on brain outcomes, with a few exceptions such as miR-144, which has vague effects (95, 96, 142). The results show that microglia are the most frequently studied cell types targeted by miRNAs after ICH, with 18 kinds of miRNAs (including the individual passenger strands) proven to have effects on them. Among them, 13 were beneficial and 5 were detrimental to neurological functions. They modulate the microglia polarization state or its signaling or both of these functions. Some of these act through classical inflammatory signaling pathways, such as the NF-κB and TLR4 pathways. Some interfere with the components related to other secondary brain injuries, such as the autophagic AKT/mTOR pathway and oxidative stress, suggesting the complexity of neuroinflammation and its overlapping mechanisms with other secondary injuries (147). Therefore, it might be more practical and efficient to target neuroinflammation and other secondary brain injuries simultaneously using miRNA-based drugs, and more research on the effects of miRNAs on other brain injuries are needed. Apart from microglia, leukocytes and astrocytes can also be regulated by miRNAs in ways related to neuroinflammation. Leukocytes include neutrophils and mast cells, the infiltration and degranulation of which can be modulated by miRNAs. The most frequently studied molecular targets of miRNAs are the well-known NLRP inflammasome family, with NLRP3 exerting a harmful function (120) and NLRP6 having an opposing protective function after ICH (130). Eight types of miRNAs that can target at least one of them have been identified. In addition, NF-κB also contributes significantly to the regulation of post-ICH neuroinflammation by miRNAs. The Nrf2 and PI3K/AKT pathways have been reported by several studies to be targeted by miRNAs, thus affecting the course of neuroinflammation after ICH (135, 137). Of note, there are contradictory results concerning the effects of several miRNAs on their targets, such as miR-144 modulation of the mTOR autophagic pathway and of neurological functions (95, 96, 142). Further research is required to verify these uncertain effects.

**Table 1:** Effects of microRNAs on immune cells, inflammatory molecules, and pathways after ICH.

Immune cells				
Cell type	Subtype	microRNA	Target	Reference
**Microglia**	(+) (apart from polarization)	miR-21/miR-146a, miR-181b, miR-7, miR-367, miR-181c, miR-26a, miR-183-5p	TLR4↓, IRAK4↓, MLL1↓, NF-κB↓, HMGA2↓, HO-1↓	(81, 83–86, 88, 146)
	(-) (apart from polarization)	miR-222, miR-144, miR-23b, miR-181a	ITGB8↓, autophagy (AKT/mTOR)↓or↑, IPMK↓, monocytes, IL-8	(94–97, 100)
	Affect MG polarization	miR-146a-5p (BMSCs-Exo), miR-34a-5p, miR-494, miR-124, miR-367, Let-7a, miR-575	IRAK1↓, NFAT5↓, Klf4↓, Nrdp1/CEBP-β↓, CEBP-α↓, CKIP-1↓, PTEN↓	(89–93, 98, 99)
**Astrocytes**		miR-146a-3p, miR-155	PI3K/Akt↑/AQP4↓, SOCS-1↓	(88, 89, 106)
**Leukocytes**	Neutrophil	let7c, miR-183-5p, miR-146a-5p (BMSCs-Exo)	IGF1R↓, HO-1↓	(112)
	Mast cell	miR-125b-2-3p	IRE1α/Lyn kinase↓	(103, 104)
**Lymphocytes**		–	–	–
**NK cells**		–	–	–
				
Molecules and pathways				
Molecule/pathway		microRNA	Targets	References
**NF-κB**		miR-195, miR-146a, miR-93, miR-140-5p, miR-146a, miR-367, miR-181c, miR-139	NF-κB↓, TLR4/NF-κB↑, TLR4/MyD88/TRIF/NF-κB↓, TRAF6/NF−κB↓, IRAK4↓, MLL1↓, Nrf2↑/NF-κB↓	(84, 85, 114–116, 118, 125, 138)
**NLRP**		miR-223, miR-152, miR-194-5p, miR-124-3p, miR−183−5p (BMSC-EV), miR-331-3p	NLRP3↓, TXNIP/NLRP3↓, TRAF6/NLRP3↓, PDCD4/NLRP3↓, NLRP6↓	(120, 121, 123, 124, 126, 130)
**PI3K/AKT**		miR-181b, miR-146a-3p	PI3K/AKT↑	(103, 135)
**Nrf2**		miR-155, miR-139	BMAL1/Nrf2↓, Nrf2↑/NF-κB↓	(137, 138)
**Other**		miR-132	AChE↓/ACh↑	(139)
		miR-590-5p	Peli1↓	(140)
		miR‐21‐5p	p-ERK/HO-1↓	(141)
		miR-155	VEGF↑	(145)
		miR-144/451	FoxO3/14-3-3↓	(142)
		miR-23a-3p	NFE2L2↓	(143)
		miR-155	lncRNA Mtss1↓	(144)
		miR-340-5p	PDCD4	(127)

↑ indicates that activity increases with the elevation of miRNA, and ↓ indicates that activity decreases with the elevation of miRNA. (+) indicates positive therapeutic effects after ICH, and (−) indicates negative therapeutic effects after ICH

Alleviation of inflammatory injury in the brain can be achieved by reducing the injurious effects and promoting the reparative effects of neuroinflammation. miRNAs play roles in both pro-inflammatory and reparative reactions as discussed above, and have multiple targets, making them flexible targets with which to modulate brain inflammation (148). In addition, it modulates neuroinflammation by functioning in both cellular and intercellular ways. On the one hand, abnormally expressed intracellular miRNAs can regulate cellular inflammatory cascades by targeting molecules and pathways involved in immune responses (74, 149). On the other hand, miRNAs can participate in intercellular communications, such as neuronal-glial interactions, by being released through EVs such as exosomes (150–152). For example, dysregulated miRNAs and their precursors have been found to be released by neural cells through exosomes, a prominent type of EV, and delivered to bystander cells, causing dysfunction and dissemination of brain injury (153). In short, miRNAs can have different influences in specific temporospatial situations, with shared or unique features, being detrimental or restorative, working at the single-cell level or whole- brain level, or anywhere in between. In the future, a better understanding of the regulation of neuroinflammation by miRNAs may help achieve precise and efficacious therapeutics that are tissue-specific (such as those specific to different hematoma locations, or gray or white matter), cell-specific (such as different functionality in microglia, astrocytes, and neurons), and time-dependent (such as modulating microglia in different inflammatory stages) (36, 154). In return, manipulating miRNAs may increase the understanding of the pathophysiological mechanism of ICH.

Of note, although the regulation of protein expression by miRNAs is essentially fine-tuning, it has some unique characteristics that make it a superior CNS drug target. First, it can regulate functionally critical molecules in pathways that may have a significant impact. In addition, since a miRNA can target multiple mRNAs, it may modulate multiple genes and have multi-molecular or even multi-pathway effects, making it efficient in treating diseases that have complicated compensatory mechanisms such as ICH (155). Apart from that, endogenous miRNAs commonly produce rapidly and degrade slowly, thus lasting a long time in human cells (the median half-life is more than 10 h), adding to the feasibility of exogenous drug intervention (156). Moreover, since miRNAs can remain either intracellular or be released by extracellular excretion, they may have the ability to target both “non-druggable” intracellular targets and intercellular communication. These unique features make miRNAs potential therapeutic targets for ICH.

### 5.2 microRNA-based therapeutics

miRNA-based therapeutics have been explored for a variety of diseases, including CNS diseases (157–160). As early as 2004, viral vectors expressing miR-181, miR-223, and miR-142 were found to modulate lymphoid differentiation of S17 bone marrow stromal cells *in vitro* (161). Since endogenous miRNA can be modulated by genetic, epigenetic, post-translational modification, competing endogenous RNA sponging and other ways, theoretically many processes in the biogenesis and functioning of miRNA can be interfered with (15, 162, 163). Examples include diverse isomiR formation, pre-miRNA editing, non-templated nucleotide addition, miRNA turnover, AGO phosphorylation, intracellular and extracellular trafficking, and subcellular functioning. However, only a few have been tested in basic research and clinical trials. These include miRNA mimics, antimiRs (known as antagomirs when conjugated to cholesterol), miRNA sponges, Tough decoy, miRNA-masking antisense oligonucleotides (ASOs), short hairpin RNAs, peptide nucleic acids, and small-molecule inhibitors. Among them, the most frequently studied types are miRNA mimics and antimiR (164, 165). The miR mimic is double-stranded and is similar to duplex siRNA, while the antimiR is more common than miR mimics because of its relatively simple single-stranded structure, which is similar to that of the widely studied ASOs. Many strategies for building antimiR drugs have been adopted from ASO drugs. Compared with other nucleotide-based drugs, miRNA-based drugs have the unique feature of targeting multiple molecules and pathways simultaneously (166). If used appropriately, it may be favorable in a complex diseased milieu, such as inflammation, where many pathways are interrupted.

Some miRNA drugs have been tested in clinical trials. For instance, antimiR103/107 RG-125 (AZD4076) was tested for the treatment of type II diabetes and nonalcoholic fatty liver disease; antimiR-122 Miravirsen (SPC3649) was tested for the treatment of HCV infection (167); antimir-92a (MrG-110) was tested for the treatment of angiogenesis and wound healing (168); and antimir-21 (rG-012) was tested for the treatment of Alport syndrome (169). As for mimics, the miR-16 mimic Mesomir-1 has been tested in phase I clinical trials for malignant pleural mesothelioma and non-small cell lung cancer, and the miR-29 mimic Remlarsen (MRG-201) was tested in a phase II trial for keloids (170). In the brain, the pri-miR-451 backbone rAAV5-miR-HTT (AMT-130) (AAV, adeno-associated virus), a drug for treating Huntington’s disease by one-time MRI-guided stereotaxic intrastriatal infusion, is currently in phase I/II clinical trials.

miRNA sponges, which contain multiple binding sites to trap and sequester miRNA, and miRNA-masking ASOs have not yet been applied in clinical settings. Small molecules that can target miRNAs, such as peptide nucleic acids and small-molecule inhibitors have seldom been reported (171). Several newly discovered small molecules that target miRNAs have been discovered by phenotypic screening instead of by mechanism-based methods (172). They have been found to target RNA’s multiple closely packed helices (which is the current frontier of progress; these small molecules are exemplified by linezolid, ribocil, branaplam, and SMA-C5), RNA’s irregular secondary structures, or RNA’s triplet repeats, which are all complicated RNA structures (173). Since the specific structure of RNA is still not well understood, the manipulation of RNA function by these molecules lacks more precise elaboration.

### 5.3 Procedures to develop microRNA-based therapeutics for ICH

**Figure 3:**
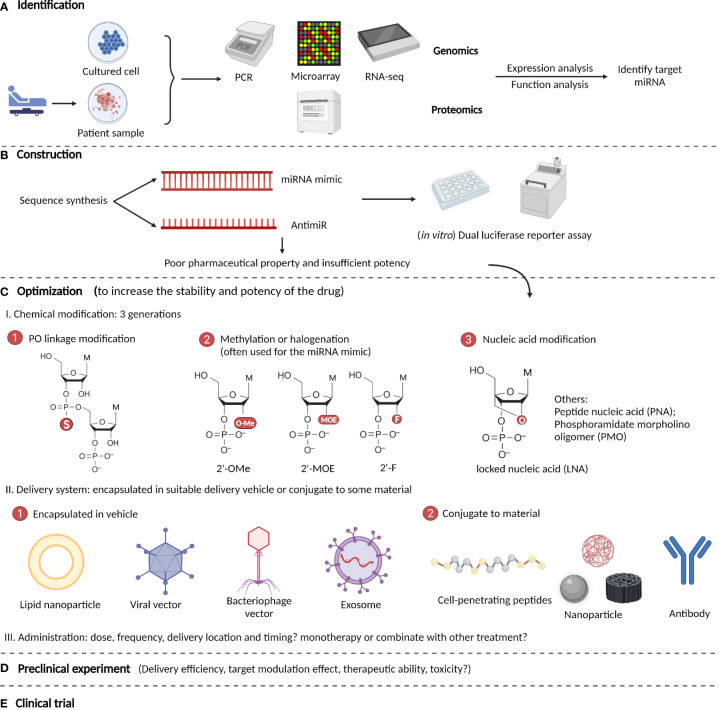
Procedures to develop microRNA-based therapeutics for ICH. **(A)** Identify target miRNA by genomic or proteomic expressional and functional analyses. **(B)** Construction of miRNA-based drugs (most are miRNA mimics and antimiRs) and performance of preliminary *in vitro* verification experiment such as dual luciferase reporter assays. **(C)** Optimization of the drug’s pharmacological properties *via* chemical modifications and optimizing the delivery systems and administration patterns. **(D)** Rigorous preclinical experiments are performed to test the drug’s potency and efficiency in ICH. **(E)** Clinical trials are performed to examine the drug’s curative efficiency, suitable administration patterns, and toxicity or side effects.

The development of miRNA-based therapeutics is a rigorous procedure that includes mainly five steps: identification, construction, optimization, preclinical tests, and clinical trials (174). The optimization step includes the modification, delivery, and administration of a drug. After the target miRNA that exerts its function is identified by genomic or proteomic expression and function analyses, an interventional drug is constructed based on its sequence and intended effects (175). The most commonly used drugs are miRNA mimics and antimiRs, which are both synthetic nucleotides that are double-stranded and single-stranded respectively. After construction, preliminary *in vitro* experiments such as dual-luciferase reporter assays are performed to verify the effects of the drug on its targets. If it meets expectations, the drug is then optimized in structure. Because the original oligonucleotides are unstable and easily degraded in the body, they require chemical modifications and appropriate delivery systems (176). The modification methods have evolved through three generations: phosphodiester (PO) linkage modification, methylation or halogenation, and nucleic acid modification. For delivery, the drug can be encapsulated in suitable delivery vehicles, or conjugated to some materials, such as nanoparticles (177). After optimization, rigorous preclinical experiments are started, and indicators such as delivery efficiency, target modulation effect, therapeutic ability, and toxicity are tested both *in vitro* and *in vivo* (178). Finally, if the drug exhibits satisfactory effects in preclinical studies, it should be tested in clinical trials with four stages to examine its curative efficiency, suitable administration patterns, and toxicity or side effects for clinical use (179). Here, we summarize the main procedures for developing miRNA-based therapeutics for ICH (**Figure 3**).

## 6 Limitations and future directions

Although miRNA-based therapeutics have been explored in clinical trials for many diseases, clinical trials for ICH are lacking. This is probably due to limitations and technological hindrances in studying the role of miRNAs in neuroinflammation. Some are related to the experimental process, while others are related to current research methods. To gain a complete understanding of the function of miRNAs and develop them as therapeutic targets for ICH, these limitations should be addressed in the future.

### 6.1 Broader mechanism studies

Neuroinflammation is a complex process that intrinsically interacts with many other secondary brain injury mechanisms such as programmed cell death, phagocytosis of hematoma and brain debris, cerebral edema, toxic product clearance, and oxidative stress, making it difficult to elucidate the explicit pathogenesis of neuroinflammation. Therefore, it is necessary to study the functions of miRNAs in other secondary brain injuries at the same time and to consider their interaction with the course of neuroinflammation. In addition, one miRNA may have multiple targets, and different miRNAs may have the same target, thus forming a complicated miRNA-inflammation network (123, 124). Moreover, different miRNA targets may have shared downstream mediators. Many overlapped mechanisms occur after miR-based drug administration, including microglial polarization, neutrophil infiltration, cytokine changes, and activation of classical molecular pathways such as the NF-κB pathway. Therefore, there may be extra or accessory mechanisms that are revealed in some studies; however, researchers may ignore these based on the study topic at hand. For example, in a study in which microglia were reported to be modulated by certain miR-based drugs, other inflammatory components such as astrocytes and leukocytes may have also been affected (180). Therefore, broader mechanistic studies are needed to elucidate the exact functions of miRNAs in post-ICH neuroinflammation.

### 6.2 Animal models

There are some limitations to study the effects of miRNAs on neuroinflammation, and some of these are manifested in the analyzed literature and are related to the experimental process. First, all existing studies used animal and *in vitro* cell models, both of which are preclinical models that may have interspecies differences from human beings in terms of reactions. Second, the animals used in most studies were mainly male rats and male mice aged 8–10 weeks. Since it has been reported that aging can cause an inflammatory microenvironment (181) and that sexual hormones might modulate inflammatory responses (182), age and sex can be confounders in these types of experiments and should be considered in future research. Third, ICH in humans is always accompanied by comorbidities such as hypertension and diabetes. Among the studies discussed, only one study used rats with diabetic intracerebral hemorrhage (126). The lack of an equivalent disease animal model related to human diseases will decrease the reliability and reference value of these studies. To develop meaningful disease models, the pathogenesis of ICH with subdivisions of different causes, locations, magnitudes, and timings needs to be clarified.

### 6.3 High-resolution observations

Apart from the limitations mentioned above, there were limitations related to the targets of the experiments. Most miRNA targets in animal models have been detected by tissue-based ensemble analyses. Therefore, their explicit positions and functions at the cellular and subcellular levels remain unknown. For example, any given miRNA may have cell type-specific actions and subcellular locations. These are important for understanding the function of miRNAs and require further investigation. Newly developed technologies might help to answer the questions, such as flow cytometry, single-cell RNA-sequencing, translocator protein radiotracer, genetic fluorescence labeling, and cell heterogeneity profiling (183–185). Recently, *in situ* single-molecule visualization was used to observe each step of the miRNA function process in cells (59). By observing each step of miRNA silencing actions at the same time, there are some novel discoveries regarding miRNA silencing mechanisms. This study provides a new framework for studying the function of miRNAs at a single-molecule resolution, with spatiotemporal specificity. Besides, conditional gene-deficient models are emerging, making it possible to study highly involved pathways and trace certain molecules in the post-ICH brain milieu. For example, one study used genetically knockout mice lacking the miRNA144/451 cluster to study its effects on brain function after ICH (142). Notably, since many miRNAs are present in families, genetically deleting one of them often leads to a weak phenotype, while gain-of-function studies show obvious phenotypes.

### 6.4 Timing and interplay

Elucidating the timing and interplay of the cellular and molecular components that are under the influence of miRNAs in the brain is difficult yet indispensable to explain the pathogenesis of post-ICH neuroinflammation and remains to be fully understood. This includes how the pathophysiological mechanisms act in a chronological sequence, their degree of importance, their balance between damage and repair, and the precise time their phenotypes transform if exist (12, 24). Solving these questions relies on a deeper understanding of the intercellular interactions among various immune cell types, which may be mediated partly by miRNAs secreted as extracellular vesicles (186, 187). A virus-based barcoding system called rabies barcode interaction detection followed by sequencing (RABID-seq) was recently developed to observe cellular interactions *in vivo*. Using this system and simultaneous transcriptomic analysis at the single-cell level, researchers discovered that microglia can modulate astrocyte reactions in experimental autoimmune encephalomyelitis by expressing Sema4D and Ephrin-B3, implicating the utility of this method in elucidating cell interactions (188). Finally, the relative scarcity of the literature and the researchers’ selection preference may add bias to the reliability of the results regarding the function of molecules in post-ICH inflammation.

## 7 Concluding remarks

Neuroinflammation is a major contributor to secondary brain injury and is associated with the clinical prognosis of ICH. miRNAs are distinctive molecules with unique expression profiles and have important effects on neuroinflammation after ICH. Preclinical studies have shown that they regulate diverse inflammatory components, including immune cells, inflammatory molecules, and pathways within the brain, and can have positive or negative effects on brain function after ICH. In fact, their multi-targeting property makes them potential targets to reverse the complex, disordered brain milieu where multiple pathways are disrupted. This finding suggests the possibility of developing miRNA-based therapeutics for ICH. Although preclinical studies on miRNA function in ICH are numerous, there have been no clinical trials of related therapeutics. This is likely due to limitations in studying their function. In the future, to develop new miRNA-based therapies for ICH, broader mechanistic studies are needed to achieve a better understanding of the characteristics of miRNAs, their temporospatial specific functions, their interactions with brain components, and the network they form to regulate overall neuroinflammation. Additionally, meaningful preconditioned disease models are required. Newly developed technologies, such as single-cell, single-molecule, and cellular interaction study methods may help. With the further elucidation of miRNA-inflammation mechanisms and advancements in pharmaceutics, effective miRNA-based therapies for treating ICH can more easily be developed.

## Author contributions

All authors have made a substantial, direct and intellectual contribution to the work, and approved it for publication.

## Funding

This study was supported by the National Key R&D Program of China (2018YFC1312600, 2018YFC1312603), and the Key R&D Program of Zhejiang Province (2022C03133).

## Acknowledgments

Some of the figures were created using BioRender.

## Conflict of interest

The authors declare that the research was conducted in the absence of any commercial or financial relationships that could be construed as a potential conflict of interest.

## Publisher’s note

All claims expressed in this article are solely those of the authors and do not necessarily represent those of their affiliated organizations, or those of the publisher, the editors and the reviewers. Any product that may be evaluated in this article, or claim that may be made by its manufacturer, is not guaranteed or endorsed by the publisher.
